# The Role of Severe Acute Respiratory Syndrome (SARS)-Coronavirus Accessory Proteins in Virus Pathogenesis

**DOI:** 10.3390/v4112902

**Published:** 2012-11-07

**Authors:** Ruth McBride, Burtram C. Fielding

**Affiliations:** 1 Anatomy Cluster, Department of Medical Biosciences, Faculty of Natural Sciences, University of the Western Cape, Private Bag X17, Modderdam Road, Bellville, Western Cape, 7535, South Africa; Email: rmcbride@uwc.ac.za; 2 Molecular Biology and Virology Laboratory, Department of Medical Biosciences, Faculty of Natural Sciences, University of the Western Cape, Private Bag X17, Modderdam Road, Bellville, Western Cape, 7535, South Africa

**Keywords:** SARS-coronavirus, accessory proteins, open reading frames, respiratory disease

## Abstract

A respiratory disease caused by a novel coronavirus, termed the severe acute respiratory syndrome coronavirus (SARS-CoV), was first reported in China in late 2002. The subsequent efficient human-to-human transmission of this virus eventually affected more than 30 countries worldwide, resulting in a mortality rate of ~10% of infected individuals. The spread of the virus was ultimately controlled by isolation of infected individuals and there has been no infections reported since April 2004. However, the natural reservoir of the virus was never identified and it is not known if this virus will re-emerge and, therefore, research on this virus continues. The SARS-CoV genome is about 30 kb in length and is predicted to contain 14 functional open reading frames (ORFs). The genome encodes for proteins that are homologous to known coronavirus proteins, such as the replicase proteins (ORFs 1a and 1b) and the four major structural proteins: nucleocapsid (N), spike (S), membrane (M) and envelope (E). SARS-CoV also encodes for eight unique proteins, called accessory proteins, with no known homologues. This review will summarize the current knowledge on SARS-CoV accessory proteins and will include: (i) expression and processing; (ii) the effects on cellular processes; and (iii) functional studies.

## 1. Introduction

Prior to the outbreak of severe acute respiratory syndrome (SARS) caused by SARS human coronavirus (HCoV) in 2003 [1,2,3], only two human coronaviruses, HCoV-OC43 and HCoV-229E, were known [4,5]. In the following two years, two additional coronaviruses were identified, namely HCoV-NL63 [6] and HKU1 [7]. Then, very recently, in September 2012 another novel coronavirus was identified in two patients, of which one died [8]. Not much is known about this latest coronavirus and studies are underway to understand the virus better. Only HCoV-OC43, HCoV-229E, HCoV-NL63 and HKU1 are known to continuously circulate in the human population [9]. The International Committee for Taxonomy of Viruses (ICTV) recently reported that the three traditional coronavirus groups have been replaced by four genera, namely Alpha-, Beta-, Gamma- and Deltacoronaviruses [10].

**Figure 1:**
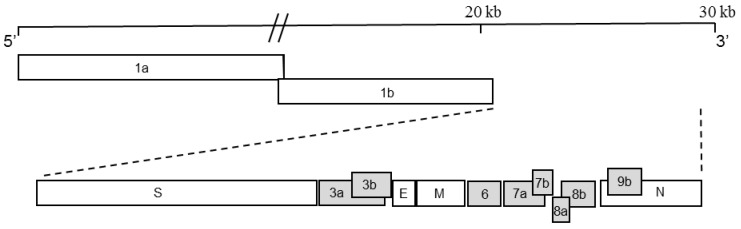
Organization of severe acute respiratory syndrome coronavirus (SARS-CoV) genome showing the position of the accessory genes. The eight accessory open reading frames (ORFs) (grey shaded boxes) encode for ORFs 3a, 3b, 6, 7a, 7b, 8a, 8b and 9b. The ORFs encoding for the replicase genes (ORF 1a and ORF 1b) and structural genes, spike (S), membrane (M), envelope (E) and nucleocapsid (N) are also shown (not drawn to scale).

SARS eventually spread to more than 30 countries in 2003, infecting ~8000 people with a mortality rate of ~10% [11]. The etiological agent was eventually identified as the SARS human coronavirus (SARS-CoV) [12]. The virus has been identified as an enveloped, positive-sense RNA virus containing a ~27 kbs genome that encodes for proteins expressed from full-length and subgenomic (sg) mRNAs [13]. In addition to the replicase and structural open reading frames (ORFs), the SARS-CoV genome contains eight ORFs encoding for accessory proteins with no known homologues (Figure 1). These group-specific accessory proteins are interspersed among the structural genes at the 3’-end of the genome.

Coronavirus accessory proteins have been shown to be dispensable for growth *in vivo* and *in vitro* [14,15,16,17,18]. On the other hand, deletion of the nonessential genes from the mouse hepatitis virus (MHV) genome results in attenuation of the virus when inoculated into the natural hosts [15], indicating a possible *in vivo* function. Continuous passage of infectious bronchitis virus (IBV) in cell culture, results in mutations in the IBV 3b gene that confers a growth advantage to the virus when cultured in cells and chicken embryos. These mutations also increase the virulence of the IBV in the chicken embryos [19]. Reports to date make it clear that the CoV accessory proteins are not essential for virus replication *in vitro*, but since these genes are maintained in the virus genomes under selective pressures, they have to confer a biological advantage to the virus in the natural environment of the infected host [20].

Although recent evidence shows that the SARS-CoV accessory genes are expressed in the host during infection, their functions remain somewhat obscure. Currently, there are a range of proposed functions for these accessory proteins, including modulation of viral pathogenicity and replication, as well as acting as cell death inducers and interferon (IFN) antagonists, to name a few. In this review the current knowledge on SARS-CoV accessory proteins (ORFs 3a, 3b, 6, 7a, 7b, 8a, 8b and 9b) will be summarized and will include: (i) expression and processing; (ii) the effects on cellular processes; and (iii) functional studies. 

## 2. SARS Accessory Proteins

### 2.1. ORF3a and ORF3b

SARS-CoV 3a protein, also referred to as ORF3 [21], X1 [22], ORF3a [23] and U274 [24], was the first of the SARS-CoV accessory proteins to be characterized. ORF3a, located at the 3´-end of the SARS-CoV genome between the S and E genes, encodes for a 274 amino acid (a.a.), 31 kDa accessory protein [21,22,25,26]. The ORF3a N-terminal ectodomain (a.a. 1-34), contains three transmembrane regions (a.a. 35-56; 77-99; 103-125), and is separated from the C-terminal endodomain (a.a. 209-264) by a central region containing a cysteine-rich domain (a.a 127-133), a YxxΦ domain (a.a. 160-163; where x is any a.a. and Φ is an a.a. with a bulky hydrophobic side-chain) and a diacidic domain (a.a. 171–173) [24]. 

Although indirect immunofluorescence detects ORF3a partly in the rough endoplasmic reticulum [27], it is mainly found perinuclearly in the Golgi complex [24,27,28]. Antibodies to ORF3a also stain the plasma membrane [24,27,29], where it is either secreted out of the cell [30,31] or undergoes endocytosis [24]. Following ORF3a translation, the diacidic domain ensures that the protein is efficiently exported from the endoplasmic reticulum [32] to the Golgi apparatus, where ORF3a undergoes posttranslational modifications in the form of O-glycosylation [33] before being inserted into the plasma membrane. Transport of ORF3a to the cell surface requires two protein sorting and tracking signals, the centrally located YxxΦ and diacidic motifs [24]. Once in the plasma membrane, the side chains of cysteine in the cysteine rich domain allow ORF3a to form homo- and heterotetramers, which cluster together to form a potassium-permeable ion channel [34]. In transfected cells, co-immunoprecipitation experiments have shown that membrane-bound ORF3a interacts with structural proteins S, M and E, as well as with SARS-CoV ORF7a, another accessory protein [24,27,35,36].

The presence of ORF3a in the cytoplasm of infected cells has been confirmed through immunohistochemical analysis of tissue biopsies and/or autopsies of SARS-CoV-infected patients. In lung sections, the expression of ORF3a is restricted to the cytoplasm of pneumocytes and to multinucleated giant cells, a rare feature of the lung pathology in SARS [37]. Moreover, sections taken from the small intestine show ORF3a localizes to the cytoplasm of surface enterocytes [37]. The co-localization of ORF3a together with the SARS-CoV genome in the cytoplasm of pulmonary pneumocytes and small intestinal enterocytes is not totally unexpected as the lungs and small intestine are primary targets of SARS-CoV infection. The lung and small intestines are, however, not the only tissue targets for SARS-CoV. One of the cellular receptors for SARS-CoV, angiotensin-converting enzyme 2 (ACE2) [38,39,40], is also expressed by a certain population of peripheral CD14+ monocytes, from which osteoclasts are derived [41]. Interestingly, the expression of ORF3a in a murine macrophage osteoclast precursor cell line enhances osteoclast differentiation, and may explain why many recovered SARS patients suffer from reduced bone density [41]. 

The first attempts to elucidate the functions of coronaviral group-specific genes involved the generation of recombinant MHV from which the group-specific genes had been deleted. These recombinant viruses could still replicate, demonstrating that these genes are dispensable for virus growth *in vitro*. The mutant viruses did, however, still lead to a significant attenuation of the MHV when inoculated into mice, suggesting that coronaviral group-specific genes play a role in virus-host interactions and therefore contribute to viral fitness [15]. Subsequently, much effort has been placed on elucidating the role of ORF3a in the life cycle of the SARS-CoV. The biological functions of ORF3a may be closely linked to those of the structural proteins with which they interact. The S protein is involved in viral assembly and packaging of coronaviruses (reviewed by [42]) and so the formation of inter-chain disulfide bonds between ORF3a and the S protein in the interior of the viral envelope may suggest that ORF3a is integrated into viral particles (reviewed by [20]). Indeed, ORF3a is incorporated into newly packaged mature SARS-CoV virions, suggesting that ORF3a is a structural protein [29,30,43]. Furthermore, the co-localization of ORF3a with M and E proteins, which are essential for viral assembly [44], suggests that ORF3a is important in SARS-CoV assembly or budding [27]. 

There seems to be some conflicting data in the literature as to the role that ORF3a plays in viral replication. In Vero E6 cells transfected with a SARS-CoV deletion mutant lacking the 3a gene, the virus still replicates as efficiently as the wild-type, suggesting a non-essential function of ORF3a in viral assembly *in vitro* [17]. Moreover, in a study that utilized small interference RNAs (siRNAs) to knockdown the expression of ORF3a in FRhK-4 cells, viral replication was also unaffected [34]. However, the transfection of Vero E6 cells with siRNAs specific to ORF3a significantly reduced the yield of progeny SARS-CoV, signifying an inhibition of SARS-CoV replication cycle in these cells [45]. This could have been a combinational effect due to the concurrent knockdown of ORF3b. In addition to a possible role in viral replication, ORF3a is likely to modulate viral release, as a study has shown that there is a significant reduction in viral release in FRhK-4 cells transfected with ORF3a specific siRNAs [34]. Taking into account that ORF3a forms a potassium-sensitive ion channel in the plasma membrane [29,34], and ion channels for viral proteins control virus entry or release [46,47], the mechanism whereby ORF3a influences viral release warrants further investigation [34]. 

ORF3a has also been shown to regulate various host cellular responses. Overexpression of ORF3a in several different cell culture models has demonstrated that it induces apoptosis [29,48,49,50], upregulates mRNA and protein levels of fibrinogen in lung epithelial cells [51], activates C-Jun N-terminal kinase (JNK) and the transcription factor nuclear factor kappa B (NF-kappaB), which is involved in the activation of pro-inflammatory genes [20,41,52] and downstream from that, up-regulates the production of pro-inflammatory cytokines and chemokines such as interleukin 8 (IL-8) and RANTES (CCL5) [52]. Taken together, these changes to host cellular homeostasis imply a role for ORF3a in the pathogenesis of SARS.

The occurrence of both apoptosis and necrosis in host cells during SARS-CoV infection suggests that the regulation of cell death is important for viral replication and/or pathogenesis (reviewed by [53]). The pro-apoptotic function of ORF3a is reliant on its ion channel activity [29] and is induced via caspase-8 and -9 dependent pathways, *i.e.* through both the death receptor- and mitochondria-mediated pathways, respectively [23,29,48]. In addition, it has recently been reported that Bax, p53 and p38 MAP kinase also play roles in ORF3a-induced apoptosis [54]. The fact that ORF3a utilizes more than one caspase pathway to trigger cell death may explain why tissue responses to SARS-CoV infection are distinct in different organs; for instance whereas the lung pathology is dominated by diffuse alveolar damage, the small intestine remains relatively intact [37].

Even though the natural animal reservoir for SARS-CoV has not been identified, the threat of another SARS outbreak is still a disturbing possibility as bats have been shown to be natural reservoirs for different SARS-like coronaviruses (SL-CoV) [55]. Like all accessory proteins, ORF3a is unique to the SARS-CoV, and shares an 83% homology to SL-CoV [56], making it a good candidate for the development of diagnostic assays, vaccines, and drugs. The immunogenic properties of ORF3a in SARS-CoV-infected patients has been shown to vary depending on: (i) whether or not the sera was collected from convalescent or recovered patients; (ii) whether or not full-length or truncated fusion proteins were used to detect immunoglobulin G (IgGs) to ORF3a; and (iii) the method used to detect these antibodies to ORF3a, namely western blot, ELISA or protein microarrays. To date, in general, IgGs to ORF3a could be detected in only ~60–73% of patient sera screened [35,57,58,59]. This could be due to frame-shift mutations within the 3a gene, resulting in a heterogeneous population of sgRNA3 transcripts in patients with acute SARS-CoV infection containing copies of both wild-type and mutant 3a genes. [24,60]. These mutant 3a genes encode for proteins with distinctively shorter N-termini than the wild-type forms [24]. 

The neutralizing activity of anti-3a sera has subsequently been assessed *in vitro*. The sera from rabbits immunized with recombinant ORF3a (a.a. 125–274) does not exhibit any significant neutralizing activity on SARS-CoV infected Vero E6 cells [59]. In another study, however, sera from rabbits immunized with a different recombinant ORF3a (a.a. 15–28) contains neutralizing antibodies that do inhibit SARS-CoV infection in Vero E6 cells [61]. These contrasting results could be explained by differences in the immunogenicity of the central region and C-terminal domain compared to the N-terminal ectodomain, respectively. Indeed, ectodomain-specific antibodies (a.a. 15–28) from convalescent-phase patient plasma recognize and destroy SARS-CoV-ORF3a-expressing cells [62]. A more recent study has showed that mice immunized with full-length 3a DNA elicit strong humoral and Th1-based cellular immune responses. The vaccine stimulated IFN-γ production mainly by CD8+ T cells and interleukin-2 (IL-2) mainly by CD4+ T cells [56]. Whether or not ORF3a will be useful as a subunit vaccine for humans, still remains to be seen though. 

ORF3b is predicted to express the 154 amino-acid 3b protein, also referred to as ORF4 [21], X2 [22] and U154 [57], via an internal ribosomal entry mechanism [21,22,25]. SARS-CoV ORF3b has been described as a multi-localized protein due to its shuttling behavior [63]. Although the protein initially accumulates in the nucleolus [64], it subsequently translocates to the outer membrane of mitochondria [63,65]. Nuclear export of other accessory proteins, namely SARS-CoV ORF9b, has also been previously reported [66] but how this spatio-temporal distribution impacts on ORF3b behavior and function is still unclear [67]. In fact, one study has reported that ORF3b is localized primarily in the nucleus with no evidence of mitochondrial localization [68]. 

Immunohistochemical analysis of tissue biopsies and/or autopsies of SARS-CoV-infected patients has failed to demonstrate the presence of ORF3b *in vivo* [37]. The antigenicity of ORF3b has also come into question as IgGs to ORF3b are detectable in SARS patient sera in some studies [69,70], but not in others [59]. To date, the presence of ORF3b in SARS-CoV-infected Vero E6 cells is the only evidence for the expression of this protein [37].

In comparison to ORF3a protein, much less is known about the function(s) of ORF3b protein. SARS-CoV deletion mutants lacking the 3b gene replicate to levels similar to those of wild-type virus in several tissue culture cell types [17], suggesting that, like ORF3a, ORF3b is dispensable for viral replication *in vitro*. Although ORF3b may not be involved in viral replication, a role for this protein in viral pathogenicity has been inferred through its ability to modulate the host innate immune response to SARS-CoV infection [68]. ORF3b has been shown to inhibit the synthesis of type 1 IFNs (IFN-alpha/beta), major components of the innate immune response; as well as with IFN signaling [67,68]. Considering that SARS-CoV-infected cells do not produce IFN [71,72], it is possible that ORF3b provides the SARS-CoV with a mechanism to subvert the IFN response, and therefore the innate immune response [68]. However, when mice are infected with mutant SARS-CoV lacking 3b genes, the deletion viruses grow to levels similar to those of wild-type virus in the lungs of BALB/c mice at day 2 post infection [17]. This demonstrates that SARS-CoV is therefore able to inhibit the host IFN response without the 3b gene [17] and emphasizes the limitations of transfection studies. Recently, SARS-CoV ORF3b was shown to modulate the transcriptional activity of the host factor, RUNX1b, indicating a physiological role in upregulating cytokines and chemokine levels during SARS-CoV infection [73]. ORF3b also induces AP-1 transcriptional activity through activation of JNK and ERK pathways [74].

SARS-CoV ORF3b has also been shown to affect cell cycle and apoptosis-regulation but again, there are discrepancies within the literature. When overexpressed in Vero E6 cells, ORF3b induces apoptosis and necrosis with an increase in caspase-3 activity [75]. Similarly, expression of ORF3b causes cell growth arrest in GO/G1 phase, inducing cell- or tissue-specific apoptosis [76]. Since data show massive necrosis in the lungs, spleen and lymph nodes, as well as increased apoptosis in cells from the spleen, liver, lung and lymph nodes of SARS patients [76,77,78], it may suggest that ORF3b contributes to these clinical observations. 

### 2.2. ORF6

ORF6, also referred to as p6 [79], is a small 63 amino acid membrane protein with no known homologues. This relatively small peptide has a ~44 amino acid residue amphipathic N-terminus and ~20 residue polar C-terminus [80]; the C-terminus contains intracellular protein sorting motifs [81]. The protein is found on cytoplasmic membranes in the endoplasmic reticulum [82] and Golgi-complex of cells overexpressing ORF6 [82,83]. The membrane topology of this protein is N-endo C-endo, with a membrane-spanning region ~36 a.a. in length [79,84]. Using immunohistochemical staining, ORF6 was detected in both SARS-CoV infected cells, as well as in the pneumocytes of the lung and surface enterocytes of the terminal ileum from 8 SARS autopsy cases [82]; the latter corresponds to the tissue-sites of infection reported by another group [37]. 

Recent work has hinted at a few possible functions for SARS-CoV ORF6. Sucrose gradient purification of SARS-CoV particles and virus capture assays show that ORF6 is associated with purified virions, with the presence of SARS-CoV proteins E, M and S sufficient for incorporation of ORF6 into virus-like particles (VLPs). When overexpressed alone in cells [79,85,86], as well as in virus infected cells [87], ORF6 is released in membrane-associated vesicular structures, most likely a subpopulation of endosomal/lysosomal vesicles [86]. The C-terminus portion ORF6 contains the sequence motif YSEL, which has been shown to target proteins for internalization from the cell membrane to endosomal/lysosomal vesicles [84]. Several groups have previously shown that coronaviruses use similar vesicular structures within infected cells, suggesting that these vesicles have a role in the viral replication cycle [86,88,89,90]. Recombinant studies with SARS-CoV lacking ORF6 remained viable *in vitro* and *in vivo* at a high multiplicity of infection (moi) [17]. In contrast, an enhancement of replication of the virus is observed at low moi, which is consistent with previous findings using recombinant studies [91]. Recombinant studies using attenuated MHV expressing SARS-CoV ORF6, show accelerated MHV replication [92] and lead to higher viral titers in both cell culture and the mouse central nervous system [83,93]. In fact, the presence of ORF6 leads to increased virulence of normally sub-lethal infections in mice [83]. The recombinant MHV studies by Tangudu and colleagues also showed that ORF6 associated with viral RNA, co-localized with replication viral RNA on cytoplasmic vesicles and increased MHV protein synthesis [20,92]. Lastly, not only did ORF6 partially co-localize with nonstructural protein 3 (nsp3), a marker for virus replication complexes [79], it also interacted with nsp8, another replicase protein [87], pointing to a putative role for ORF6 in replication. Collectively, these results provide strong evidence that ORF6 plays a role in enhancing SARS-CoV replication and pathogenicity by interacting with membrane-bound replication and assembly machinery [84].

Interestingly, overexpression of ORF6 in CHO cells induces DNA synthesis, of which the biological significance is still not clear [82]. However, this was done in the absence of other viral proteins and needs to be verified in the context of the whole viral genome. On the other hand, ORF6 suppresses the expression levels of co-transfected expression constructs. The ability of ORF6 to interfere with the expression of co-expressed constructs has previously been linked to the blockage of nuclear translocation of these proteins [80,93]. Gunalan and co-workers also found that, even though ORF6 did not have a global effect on protein synthesis, it down-regulated the mRNA level of co-transfected nsp8. However, they linked the N-terminus diacidic cluster motif, DDEE which is similar to the putative diacidic motif DxE, which governs ER export and localization to different membranous compartments [32,86], to this suppression [86]. 

ORF6 is a type I IFN antagonist, and its expression suppresses the induction of both IFN as well as the IFN signaling pathways [68,94]. The protein inhibits IFN signaling by interacting via its C-terminal region to the nuclear import protein, karyopherin α2, thereby sequestering it in the cytoplasm and indirectly preventing movement of signal transducer and activator of transcription factor 1 (STAT1) to the nucleus [94]; the 18 a.a. N-terminus is also required for maximal inhibition [79] and this inhibition probably plays a role in the immune evasion of SARS-CoV [94]. ORF9b immunoprecipitated with ORF6 from extracts of SARS-CoV infected cells and subsequent mass spectrometry analysis reveal an interaction of the two in biologically relevant conditions. This interaction has been validated by co-localization of both proteins in the cytoplasm of SARS-CoV infected cells [95]. Taken together, findings support the hypothesis that ORF6 could be a minor structural protein and could be an important virulence factor during SARS-CoV infection *in vivo*.

### 2.3. ORF7a and ORF7b

The SARS-CoV ORF7a protein, also known as ORF8 [21], U122 [96] or X4 [22,97], is encoded by the first ORF of sgRNA7 [25,26]. The protein is a 122 a.a. long, type 1 transmembrane protein with an N terminal signal peptide sequence of 15 aa, an ectodomain of 81 aa, and a C terminus transmembrane domain containing 21 aa residues followed by a short cytoplasmic tail of 5 aa residues [98]. The ORF7a shares a 26% sequence homology with human CD62L protein, an adhesive molecule that plays important roles in immune and inflammatory responses, suggesting that ORF7a may adhere to other cells and influence their functions, such as leukocyte adhesion, migration and the inflammatory response [97]. In fact, the ectodomain bears high structural and similar sequence homology to members of the immunoglobulin superfamily, namely the D1 domains of ICAM-1 and ICAM-2 [98,99]. The ectodomain of ORF7a binds to the alpha integrin I domain of human lymphocyte function-related antigen 1 (LFA-1) on Jurkat cells, suggesting that LFA-1 may be an attachment factor or even the receptor for SARS-CoV on human leukocytes [99]. The C-terminus contains a motif (KRKTE) that typically serves as an ER retrieval signal but in the case of ORF7a, serves as a dibasic ER export signal that allows for the ER exit of ORF7a to the Golgi apparatus [96]. 

Expression of ORF7a in SARS-CoV-infected cells and lung tissue of SARS patients has been confirmed [96,97,98], but there are discrepancies in the literature as to the exact subcellular localization of the protein. It seems to localize predominantly to the intermediate compartments between the ER and Golgi apparatus [13,96,97,98,100], partially in the mitochondria [101], as well as in the cytoplasm [100]. The export of ORF7a from the ER to the Golgi apparatus is regulated via the ER export motif [96,102]. Therefore the presence of ORF7a in the ER-Golgi intermediate compartment, where coronaviruses are known to assemble [103], could explain how ORF7a becomes incorporated into purified SARS-CoV particles [31]. The interaction between ORF7a and the viral structural proteins M, E and S [31,96], which are important for viral assembly, implies that ORF7a is important in the viral replication cycle and that virion-associated ORF7a protein may exert its function early in infection [31]. ORF7a also interacts with the accessory ORF3a which itself interacts with M, E and S, suggesting that these viral proteins may form complexes in SARS-CoV-infected cells [24]. The incorporation of ORF7a in VLPs occurs however in the absence of S or ORF3a protein interaction [31]. The presence of 7a protein in the cytoplasm could explain why ORF7a is able to inhibit protein translation [13]. How this would be beneficial to the viral life cycle is unclear as the SARS-CoV relies on host cell protein synthesis to replicate viral proteins. Expressed ORF7a also interacts with human small glutamine-rich tetratricopeptide protein [100], human lymphocyte function-associated antigen 1 (LFA-1) on Jurkat cells and with human Ap4A-hydrolase [104].

ORF7b is encoded by the second ORF of sgRNA7 and may be expressed by “leaky scanning” of ribosomes [25]. This protein has been partially characterized [105] and is a 44 amino-acid long integral membrane protein that is localized to the Golgi compartment in SARS-CoV-infected cells [102]. The protein is found in association with intracellular virus particles and also in purified virions [102].

To date, there is very little experimental evidence to support a role for ORF7a or ORF7b in the replication of SARS-CoV. Monkey Vero E6 cells transfected with SARS-CoV deletion mutants lacking the 7a and 7b genes still release virions at levels similar to those of wild-type virus [102,106] and virion morphology remains unchanged [107]. In airway epithelial cultures generated from Golden Syrian hamster tracheas, SARS-CoV replication titers are comparable to infected human cultures [106]. Transgenic mice expressing the SARS-CoV receptor ACE2, one of the receptors for SARS-CoV, also still release virions when infected with mutant SARS-CoV [107]. Moreover, studies in immunosuppressed Syrian golden hamsters, an animal model for the study of SARS-CoV, have shown that infection with mutant SARS-CoV bearing deletions in gene 7 does not result in significant changes to replication kinetics, tissue tropism, morbidity or mortality [102]. One study utilizing siRNAs targeting specifically ORF7a and ORF7b has, however, demonstrated that the silencing of these proteins significantly reduces the yield of progeny virus [45]. The 70% decrease in progeny virus is not due to a disruption of the full-length genomic mRNA but reflects inhibition of both sgRNA 7 and 8, resulting in the subsequent knockdown of ORF7a, -7b, -8a and -8b [45]. Moreover, the limitations of *in vitro* studies must be taken into consideration. For example, although the 7b gene of feline coronavirus is easily lost upon virus adaptation to cell culture, it is strictly maintained in naturally occurring strains and its loss is correlated with reduced virulence [108]. The importance of ORF7a and ORF7b during the replication cycle of SARS-CoV therefore still remains uncertain. 

ORF7a may be important for viral host interactions and therefore contribute to viral pathogenesis *in vivo* by inducing inflammatory responses in SARS. Like ORF3a, ORF7a activates NF-kappaB and the IL-8 promotor [52], and downstream from that, augments the production of pro-inflammatory chemokines such as IL-8 and RANTES [52]. ORF7a may also play a role in the pathogenesis of SARS-CoV by way of the protein’s contribution to virus-induced apoptosis. Several studies have shown that overexpression of ORF7a induces apoptosis in a caspase-3-dependent manner [101,109] as well as independently via p38 MAPK [13]. Although deletion of gene 7 does not alter markers of early apoptosis or activation of caspase 3, viruses with gene 7 disruptions are not as efficient as wild-type virus in inducing DNA fragmentation, an apoptotic feature in later stages of infection [102]. Overexpression of Bcl-XL protein, a member of the pro-survival Bcl-2 family, blocks ORF7a-induced apoptosis in 293T cells, suggesting that ORF7a regulates host cell apoptosis at or upstream of the Bcl-2 family in the apoptotic pathway [101]. In addition, ORF7a forms complexes with Bcl-XL protein and binds to other pro-survival Bcl-2 family members, indicating that it may induce apoptosis by interfering with the pro-survival function of Bcl-XL protein [101]. It must be kept in mind though that ORF7a is not the only apoptosis-inducing factor during SARS-CoV infection (review by [53]) and that the actual levels at which ORF7a is expressed in infected tissues is still unknown bringing into the question the validity of the overexpression data in the *in vivo* context.

Although ORF7a is expressed in SARS-CoV-infected cells [96,97] and in lung tissue of SARS patients, specifically in the bronchial epithelial cells, peripheral blood erythrocytes and leukocytes [37], it does not display strong immunogenicity. Protein microarrays fail to detect antibodies to ORF7a in SARS patient sera [59] and SARS patient sera has low to non-specific antibody responses to ORF7a epitopes when tested on a peptide chip platform [70]. The selected ORF7a epitopes are therefore poorly immunogenic, and so ORF7a is not likely to serve as a potential marker for SARS-CoV infection or be important for vaccine development. The ORF7b protein is likely to be expressed *in vivo* because anti-7b antibody has been detected in SARS convalescent patient serum [69]. 

### 2.4. ORF8a and ORF8b

Whereas the SARS-CoV isolated from humans infected during the peak of the SARS epidemic, encodes two accessory proteins termed ORF8a and ORF8b, the SARS-CoV isolated from animals and some early human isolates, encodes for a single 122 a.a. protein, ORF8ab. Interestingly, this difference is due to a 29 nucleotide deletion in the mRNA8 of SARS-CoV from some animal species [110,111,112,113,114], that splits ORF 8 into ORF8a and ORF8b, encoding 39- and 84-residue polypeptides, respectively [110]. In fact, larger deletions were reported in sequenced ORF8 genes from a cluster of viruses that were isolated from humans later during the SARS epidemic [111,115]. 

Vaccinia virus expressed SARS-CoV ORF8ab, contains a cleavable signal sequence, which directs the movement of the precursor protein to the ER and then mediates its translocation into the ER lumen, where it is N-glycosylated on the N81 residue [110,116] and subsequently assembled into disulfide-linked homo-multimeric complexes, which remains stably in the ER [110]. This retention and accumulation of ORF8ab in the ER occurs in the absence of a recognized ER retention signal [110,117]. Not only do *in vitro* and *in vivo* studies show that ORF8ab is ubiquitinated, but both ORF8a and ORF8b also binds to monoubiquitin and polyubiquitin, suggesting the potential involvement of these proteins in the pathogenesis of SARS-CoV [116]. ORF8ab interacts with SARS-CoV proteins S, M, ORF3a and ORF7a [118], all of which have been shown to be structural proteins. 

Since ORF8a is too small for its signal sequence to function, it is most likely directly released from the ribosome and remains in the cytosol in its precursor form [110]. Antibodies to ORF8a have been detected in a small number of SARS patients, proving that the protein is indeed expressed during virus infection [119]. This group also reported that ORF8a not only enhances viral replication, but also induces apoptosis through a mitochondria-dependent pathway [119]. 

When overexpressed, the 9.6 kDa ORF8b localizes to both the nucleus and cytoplasm of Vero E6 and CHO cells [120]. In another study, however, ORF8b was not detected in SARS-CoV-infected cells or when expressed from mRNA's mimicking mRNA8. However, after it was cloned immediately downstream of a T7 promoter, a soluble, unmodified and monomeric ORF8b protein was expressed in the cytoplasm, which was extremely unstable and rapidly degraded [120]. Similarly, in the absence of the ORF8a region, ORF8b undergoes rapid degradation by proteasomes [116]. ORF8b down-regulates the expression of SARS-CoV E, a process that could be inhibited by the addition of proteasome inhibitors [116]. For this reason, E is not detectable in SARS-CoV-infected cells that are expressing high levels of ORF8b [118]. Similarly to ORF6, overexpression of ORF8b in mammalian cells also induces DNA synthesis. However, co-expression of SARS 8b and SARS 6 did not elicit synergistic effects on DNA synthesis [120]. The biological function of this induction is still to be elucidated.

It is obvious that the 29-nucleotide deletion disrupts the proper expression of the SARS-CoV ORF8. Even though this mutation does not appear to adversely affect the survival of the virus, it has become clear that ORF8ab, -8a and -8b have different stabilities and varying functions, and could contribute differently to viral replication and pathogenesis *in vivo*. Taken together these findings suggest that ORF8ab may modulate viral replication and/or pathogenesis in a yet unknown manner. 

### 2.5. ORF9b

Bi-cistronic mRNA9 encodes for ORF9b which is translated via a leaky ribosomal scanning mechanism [121]. This 98 amino acid peptide is synthesized from an alternative complete reading frame within the N gene [37,121]. It has no homology with known proteins and, as yet, its function has not been confirmed. Both anti-9b antibody and ORF9b antigen have been detected in clinical samples from SARS patients [37,59], with the latter also detected in virus infected cells [37]. Analysis, using fluorescence microscopy of live transfected cells and indirect immunofluorescence of transfected fixed cells, shows that ORF9b is exported outside of the cell nucleus and localizes to the endoplasmic reticulum [66,122]; the authors suggest that the 46-LRLGSQLSL-54 amino acid sequence motif of ORF9b functions as a nuclear export signal (NES) [66]. More recently, it was confirmed that ORF9b interacts with the cellular protein Crm1 and gets exported out of the nucleus using an active NES; this nucleocytoplasmic export of ORF9b is linked to apoptosis [123]. Cellular CRM1 (Exportin1) protein functions as a nuclear export receptor for proteins carrying a Rev-like NES in a process that also requires the GTP bound form of the Ran GTPase [124]. The authors concluded that the interaction of ORF9b with Crm1 is essential for the proper degradation of ORF9b and blocking the nuclear export of the latter induces apoptosis. Also, ORF9b was shown to enter the nucleus in the absence of any nuclear localization signal (NLS) by passive transport, independent of the cell cycle [123]. 

The resolved crystal structure of ORF9b shows that the protein has a novel fold, a dimeric tent-like β structure and a central hydrophobic cavity involved in lipid binding. Since the protein also co-localizes with intracellular vesicles, it has been speculated that the protein has a role in virus assemble via membrane association [125]. Interestingly, recent evidence shows that ORF9b is a structural protein incorporated into both purified virions and VLPs, with efficient incorporation dependent on the co-expression of viral structural proteins E and M [121]. The interaction of ORF9b with another SARS-CoV accessory protein, ORF6, was discussed earlier in this review. 

## 3. Conclusions

Viruses generally encode for: (i) proteins functioning in the replication and transcription of the viral genome; (ii) structural proteins of the virion; and (iii) accessory proteins that enable, facilitate or modulate the infection process. These accessory proteins normally act by interfering with cellular processes or by modulating virus-host interactions at the level of the organism (Table 1). It has also been shown that these proteins are often dispensable for virus replication *in vitro*, but required for optimal replication and virulence in the natural host [110]. SARS-CoV is the prototype human CoV and has been studied extensively. In addition, SARS-CoV contains eight encoded accessory genes interspersed amongst the viral structural genes [22,25,126], the most of the known CoVs. Interestingly, these accessory genes are present in SARS-CoVs isolated from bats, civet cats, raccoon dogs, and humans, suggesting important accessory functions in a variety of hosts [55,127,128]. Evidence confirms that these accessory proteins are indeed expressed during SARS-CoV infection, but for many, their functions are yet to be determined. Five of the SARS-CoV accessory proteins (ORF3a, -6, -7a, -7b and -9b) have been identified as minor viral structural components, and others induce ion channels, act as cell death inducers and IFN antagonists (Table 1). It would be interesting to determine whether the high SARS-CoV accessory gene number correlate with the increased virulence of SARS-CoV when compared to the other known human CoVs. For this reason, the role of the accessory proteins in pathogenesis must be elucidated. 

**Table 1:** Summary of the effects of the SARS-CoV accessory proteins on cellular processes.

Gene Nomenclature	Effect on cellular processes ^¥^
ORF3a	Enhances osteoclast differentiation in murine macrophage osteoclast precursor cell line [41]
	Induces apoptosis via caspase-8 and -9 dependent pathways [29,48,49,50]; Bax, p53 and p38 MAP kinase also plays role in ORF3a apoptosis [54]
	Upregulates fibrinogen levels in lungs [51]
	Activates JNK and NK-kappaB, which is involved in the activation of pro-inflammatory genes [20,41,52]
	Upregulates the production of pro-inflammatory cytokines and chemokines such as IL-8 and RANTES [20,41,52]
ORF3b	Upregulates cytokines and chemokines by modulating the transcriptional activity of RUNX1b [73]
	AP1 transcriptional activity through activation of JNK and ERK pathways [74]
ORF6	Induces DNA synthesis [82]
	Suppresses the induction of type 1 IFN and IFN signaling pathways [68,94]
ORF7a	Induces inflammatory response by activating NF-kappaB and the IL-8 promotor [52]
	Augments the production of pro-inflammatory chemokines such as IL-8 and RANTES [52]
	Induces apoptosis [13,101,109]
ORF7b	ND ^#^
ORF8a	Induces apoptosis through mitochondria-dependent pathway [119]
ORF8b	Induces DNA synthesis [120]
ORF9b	Induces apoptosis [123]

^¥^: The effects of the accessory proteins on cellular functions in cells transfected with DNA constructs (for expressing individual accessory proteins), or in cells infected with SARS-CoV or recombinant viruses expressing the individual accessory proteins, are reported. ^#^: Not Determined.
